# Fisetin Inhibits NLRP3 Inflammasome by Suppressing TLR4/MD2-Mediated Mitochondrial ROS Production

**DOI:** 10.3390/antiox10081215

**Published:** 2021-07-28

**Authors:** Ilandarage Menu Neelaka Molagoda, Athapaththu Mudiyanselage Gihan Kavinda Athapaththu, Yung Hyun Choi, Cheol Park, Cheng-Yung Jin, Chang-Hee Kang, Mi-Hwa Lee, Gi-Young Kim

**Affiliations:** 1 Department of Marine Life Science, Jeju National University, Jeju 63243, Korea; neelakagm2012@gmail.com (I.M.N.M.); gihankavinda@yahoo.com (A.M.G.K.A.); 2 Research Institute for Basic Sciences, Jeju National University, Jeju 63243, Korea; 3 Department of Biochemistry, College of Oriental Medicine, Dong-Eui University, Busan 47227, Korea; choiyh@deu.ac.kr; 4 Division of Basic Sciences, College of Liberal Studies, Dong-Eui University, Busan 47340, Korea; parkch@deu.ac.kr; 5 Key Laboratory of Advanced Technology for Drug Preparation, School of Pharmaceutical Sciences, Zhengzhou University, Zhengzhou 450001, China; cyjin@zzu.edu.cn; 6 Nakdongggang National Institute of Biological Resources, Sangju 37242, Korea; ckdgml3735@nnibr.re.kr (C.-H.K.); blume96@nnibr.re.kr (M.-H.L.)

**Keywords:** fisetin, NLRP3 inflammasome, NF-κB, mitochondria reactive oxygen species, p62

## Abstract

Fisetin has numerous therapeutic properties, such as anti-inflammatory, antioxidative, and anticancer effects. However, the mechanism by which fisetin inhibits NLRP3 inflammasome remains unclear. In this study, we observed that fisetin bound to TLR4 and occluded the hydrophobic pocket of MD2, which in turn inhibited the binding of LPS to the TLR4/MD2 complex. This prevented the initiation of scaffold formation by the inhibition of MyD88/IRAK4 and subsequently downregulated the NF-κB signaling pathway. The result also demonstrated that fisetin downregulated the activation of the NLRP3 inflammasome induced by LPS and ATP (LPS/ATP) and the subsequent maturation of IL-1β. Fisetin also activated mitophagy and prevented the accumulation of damaged mitochondria and the excessive production of mitochondrial reactive oxygen species. The transient knockdown of *p62* reversed the inhibitory activity of fisetin on the LPS/ATP-induced formation of the NLRP3 inflammasome. This indicated that fisetin induces p62-mediated mitophagy for eliminating damaged mitochondria. Recently, the existence of inflammasomes in non-mammalian species including zebrafish have been identified. Treatment of an LPS/ATP-stimulated zebrafish model with fisetin aided the recovery of the impaired heart rate, decreased the recruitment of macrophage to the brain, and gradually downregulated the expression of inflammasome-related genes. These results indicated that fisetin inhibited the TLR4/MD2-mediated activation of NLRP3 inflammasome by eliminating damaged mitochondria in a p62-dependent manner.

## 1. Introduction

Microglia are resident myeloid-derived cells in the central nervous system [1] and are considered primary mediators of neuroinflammation [2]. The aberrant activation of microglia during infections is strongly associated with the death of neuronal cells and is induced by the excessive production of inflammatory substances, including reactive oxygen species (ROS) and proinflammatory cytokines, such as interleukin (IL)-1β, IL-18, IL-6, and tumor necrosis factor-α (TNF-α) [1]. This ultimately leads to the development of neurodegenerative diseases, including Alzheimer’s disease (AD), Parkinson’s disease (PD), and multiple sclerosis [3,4]. In particular, IL-1β is considered an important proinflammatory cytokine in the progression of inflammation related to neuronal disorder [5]. In this regard, intracellular multiprotein complexes, known as inflammasome, have been extensively investigated, because they are the major regulators that trigger the release of mature IL-1β [6]. To date, the most well-known inflammasome is the nucleotide-binding domain, leucine-rich family, pyrin domain-containing 3 (NLRP3) inflammasome [7], which comprises NLRP3, the apoptosis-associated speck-like protein containing a caspase-1 recruitment domain (ASC), and caspase-1 [8]. Although the precise molecular mechanism underlying the activation of NLRP3 inflammasome remains to be elucidated, various stimulants, including adenosine triphosphate (ATP), nigericin, pore-forming toxins, and particulate matter, are known to trigger the activation of the NLRP3 inflammasome [9]. Furthermore, the excessive production of mitochondrial ROS (mtROS) can stimulate the assembly and activation of the NLRP3 inflammasome in response to lipopolysaccharide (LPS) and ATP, which is accompanied by the depolarization of the mitochondrial membrane potential [10,11]. The excessive production of mtROS is limited by an increase in mitophagy, which involves the autophagic clearance of mitochondria that are damaged by ROS [12]. During mitophagy, damaged mitochondria are recognized by microtubule-associated protein light chain 3 (LC3), which leads to mitochondrial fusion with the autophagosomes via the PINK1-Parkin-p62-mediated ubiquitin-dependent pathway, or the BCL2/adenovirus E1B 19 kDa protein-interacting protein 3, FUN14 domain containing 1, and cardiolipin-mediated ubiquitin-independent pathway [13]. Therefore, the elimination of damaged mitochondria may attenuate the excessive production of mtROS and could serve as a potential strategy for inhibiting the NLRP3 inflammasome-mediated maturation of IL-1β [14,15].

Fisetin (3,7,3′,4′-tetrahydroxy flavone) is a flavonol that is ubiquitous in trees, fruits, and vegetables [16,17]. It has been reported that fisetin has numerous pharmacological properties, including anticancer [18], antioxidative [19], antimelanogenic [20], and anti-inflammatory [21] activities. Nevertheless, the detailed molecular mechanisms underlying the effects of fisetin on the activation of NLRP3 inflammasome remain unclear. In this study, we tried to evaluate the antiinflamamtory effect of fisetin by targeting NLRP3 inflammasome and observed that fisetin inhibits the LPS/ATP-induced activation of the NLRP3 inflammasome by eliminating damaged mitochondria.

## 2. Materials and Methods

### 2.1. Reagents and Antibody

Fisetin, 3-(4,5-dimethylthiazol-2-yl)-2,5-diphenyl-tetrazolium bromide (MTT), LPS from *Escherichia coli* O111:B4, ATP disodium salt hydrate, neutral red, and N-phenylthiourea (PTU) were obtained from Sigma-Aldrich (St. Louis, MO, USA). Fetal bovine serum (FBS), antibiotic mixture, and Dulbecco’s Modified Eagle’s Medium (DMEM) were obtained from WelGENE (Gyeongsan-si, Gyeongsangbuk-do, Republic of Korea). Antibodies against ASC (sc-22514), caspase-1 (sc-56036), p50 (sc-8414), p65 (sc-8008), LC3 (sc-376404), p62 (sc-48402), nucleolin (sc-13057), and β-actin (sc-69879) were purchased from Santa Cruz Biotechnology (Santa Cruz Biotechnology, Santa Cruz, CA, USA). Antibodies against NLRP3 (15101S) was purchased from Cell Signaling Technology (Beverly, MA, USA). Thermo Fisher Scientific (Waltham, MA, USA) and GenTex (Zeeland, MI, USA) supplied specific antibodies against IRAK (PA5-20018) and MyD88 (GTX-112987), respectively. Peroxidase labelled anti-mouse immunoglobulins (sc-16102) and anti-rabbit immunoglobulins (KO211708) were purchased from Santa Cruz Biotechnology and KOMA Biotechnology (Seoul, Republic of Korea). Alexa Fluor 647-conjugated secondary antibody was purchased from Abcam (Cambridge, MA, UK). Dako Faramount Aqueous Mounting Media was obtained from Dako (Carpinteria, CA, USA). All other chemicals were purchased from Sigma-Aldrich.

### 2.2. Cell Culture and Viability Assay

BV2 microglial cells (from E.H. Joe, Ajou University School of Medicine, Suwon, Gyeonggi-do, Republic of Korea) were cultured at 37 °C in 5% CO_2_ in DMEM supplemented with 5% FBS. The cells (1 × 10^5^ cells/mL) were pretreated with fisetin (0–20 µM) for 2 h and primed with 1 µg/mL LPS for 2 h and subsequent stimulation with 1 mM ATP (LPS/ATP) for an additional 24 h. Then, an MTT assay was performed to measure cell viability [22].

### 2.3. Analysis of Viability and Dead Cells Populations

BV2 microglial cells were pretreated with fisetin (0–20 µM) for 2 h prior to stimulation with LPS/ATP for 24 h. Then, the cells were stained with a Muse Count & Viability Kit (Luminex, Austin, TX, USA). Cell viability (%) and dead cell population (%) were measured using a Muse Cell Analyzer (Luminex).

### 2.4. Molecular Docking

The crystal structure of the Toll-like receptor 4 (TLR4)/myeloid differentiation factor 2 (MD2) complex (PDB ID: 3FX1) was obtained from RCSB protein database bank (PDB). The representation of the fisetin (PubChem CID: 5281614) structure was supplied from PubChem (http://pubchem.ncbi.gov, accessed on 8 July 2021), and canonical simplified molecular input line entry (SMILES) format (C1=CC(=C(C=C1C2=C(C(=O)C3=C(O2)C=C(C=C3)O)O)O)O) was used for molecular docking prediction. Molecular docking scores and poses were calculated in Mcule (Mcule Inc., Palo Alto, CA, USA, www.mcule.com). Binding site center was set up to X = 10, Y = 10, and Z = 10. All atoms/bonds were detected within <5 Å using USCF Chimera (Resource for Biocomputing, Visualization, and Informatics at the University of California, San Francisco, CA, USA, www.cgl.ucsf.edu, accessed on 8 July 2021). Relax constraints for hydrogen bonds was calculated by 0.4 Å and 20 degrees. All other parameters maintained the default settings.

### 2.5. Measurement of IL-1β by ELISA

BV2 microglial cells were pretreated with fisetin (0–5 µM) for 2 h. Then, the cells were stimulated with LPS/ATP for 48 h. Extracellular IL-1β was detected using an ELISA Kit (Thermo Fisher Scientific).

### 2.6. Western Blotting

BV2 microglial cells were pretreated with fisetin (0–5 µM) for 2 h and then stimulated with LPS/ATP for 18 h. Total protein was extracted using a PRO-PREP Protein Extraction Solution (iNtRON Biotechnology, Sungnam, Gyeonggi-do, Republic of Korea), and protein concentrations were determined using a Bio-Rad Protein Assay Kit (Bio-Rad, Hercules, CA, USA). Western blotting was performed to detect the expression of the indicated proteins according to our previous protocol [23].

### 2.7. Reverse Transcription-Polymerase Chain Reactions (RT-PCR) Using Mouse Specific Primers

Easy-Blue Reagent (iNtRON Biotechnology) was used to extract total RNA according to the manufacturer’s instructions. Target genes were amplified using the One-Step RT-PCR Premix (iNtRON Biotechnology). The specific primers and PCR conditions are shown in Table 1 [24].

### 2.8. Immunostaining of p65 and p62

BV2 microglial cells (1 × 10^4^ cells/mL) on 3% gelatin-coated coverslips were treated with fisetin (0–5 µM) for 2 h, and then, stimulated with LPS/ATP for 1 h. Cells were fixed with 4% paraformaldehyde and permeabilized with 0.1% Triton X-100. The cells were incubated with p65 and p62 antibodies (1:100 in 10% donkey serum) and treated with Alexa Fluor 647-conjugated secondary antibody. The nuclear counterstaining was performed using DAPI (300 nM), and the slides were mounted with Dako Faramount Aqueous Mounting Media. Fluorescence images were captured using a CELENA S Digital Imaging System (Logos Biosystems, Anyang, Gyeonggi-do, Republic of Korea).

### 2.9. Analysis of mtROS

BV2 microglial cells were treated with fisetin (0–5 µM) for 2 h and then, exposed to LPS/ATP for 1 h. After washing the cells with PBS, 2 µM MitoSOX Red (Thermo Fisher Scientific) was loaded for 10 min. Fluorescence intensities were measured by GloMax 96 Microplate Luminometer (Promega, Madison, WI, USA) and cell images were captured using a CELENA S Digital Imaging System.

### 2.10. Mitochondrial Membrane Depolarization

BV2 microglial cells were treated with fisetin (0–5 µM) for 2 h followed by stimulation with LPS/ATP for 1 h. The cells were stained using a Muse MitoPotential Kit (Luminex Corp. Austin, TX, USA), and mitochondrial membrane depolarization was measured using a Muse Cell Analyzer.

### 2.11. Transfection of p62 Small Interfering RNA (siRNA)

BV2 microglial cells were seeded at a density of 1 × 10^4^ cells/mL and transiently transfected with 10 nM *p62* siRNA (sip62) and control siRNA (siCON) duplex (Santa Cruz Biotechnology) with G-Fectin Transfection Reagent (Genolution Pharmaceuticals Inc., Seoul, Republic of Korea) according to the manufacturer’s protocol.

### 2.12. Maintenance of Zebrafish Embryo and Larvae

Zebrafish study was approved the Animal Care and Use Committee of Jeju National University (Jeju Special Self-governing Province, Republic of Korea; approval No.: 2020-0024), and all methods were carried out in accordance with the approved guideline [25]. Fertilized embryos were collected through natural spawning and cultured at 28.5 °C in E3 embryo media containing 2 mg/L methylene blue.

### 2.13. Cardiac Toxicity Evaluation

Zebrafish larvae (3 days post-fertilization) were pretreated with fisetin (0–400 µM) for 2 h prior to treatment with 5 µg/mL LPS for 2 h and subsequent stimulation with 2 mM ATP for 24 h. Each group of larvae (*n* = 20) was cultured at 28.5 °C and observed for the mortality. The heart rate of the larvae was manually counted using stereomicroscopy (Olympus, Tokyo, Japan) for two minutes and used as an indicator for the cardiac toxicity evaluations [26]. The heart rate was represented as beats/min.

### 2.14. Neutral Red Staining

Macrophage in zebrafish larvae was detected using neutral red staining 24 h after LPS/ATP treatment. Briefly, zebrafish larvae were treated in 2.5 μg/mL neutral read solution (Sigma-Aldrich) containing 0.003% PTU at 28.5 °C for 6–8 h [26]. After staining, macrophages in the brain area were observed using stereomicroscopy.

### 2.15. Isolation of Total Zebrafish mRNA and RT-PCR

Total RNA was extracted from zebrafish larvae 18 h after LPS/ATP treatment using Easy-Blue Reagent. The RNA was reverse-transcribed and amplified using a One-Step RT-PCR Kit. The specific primer sequences for *IL-1β* and *β-actin* were obtained from a previous study [27], and the sequences of *ACS*, *caspase-A*, and *caspase-B* were designed based on the NCBI gene database (Table 1).

### 2.16. Statistical Analysis

All western blots and RT-PCR bands were quantified using ImageJ 1.50i (National Institute of Health, Manassas, VA, USA, www.imagej.net, accessed on 8 July 2021) and then statistically analyzed by Sigma plot 12.0 (Systat Software, San Jose, CA, USA, www.systatsoftware.com, accessed on 8 July 2021). All data represented the mean of at least three independent experiments. Significant differences between groups were determined using a Student’s *t* test and an unpaired one-way ANOVA test with Bonferroni correction. Statistical significance was set at *** and ^###^ *p* < 0.001, ** *p* < 0.01, and * and ^#^ *p* < 0.05.

## 3. Results

### 3.1. High Concentrations of Fisetin Is Cytotoxic to BV2 Microglial Cells

**Figure 1:**
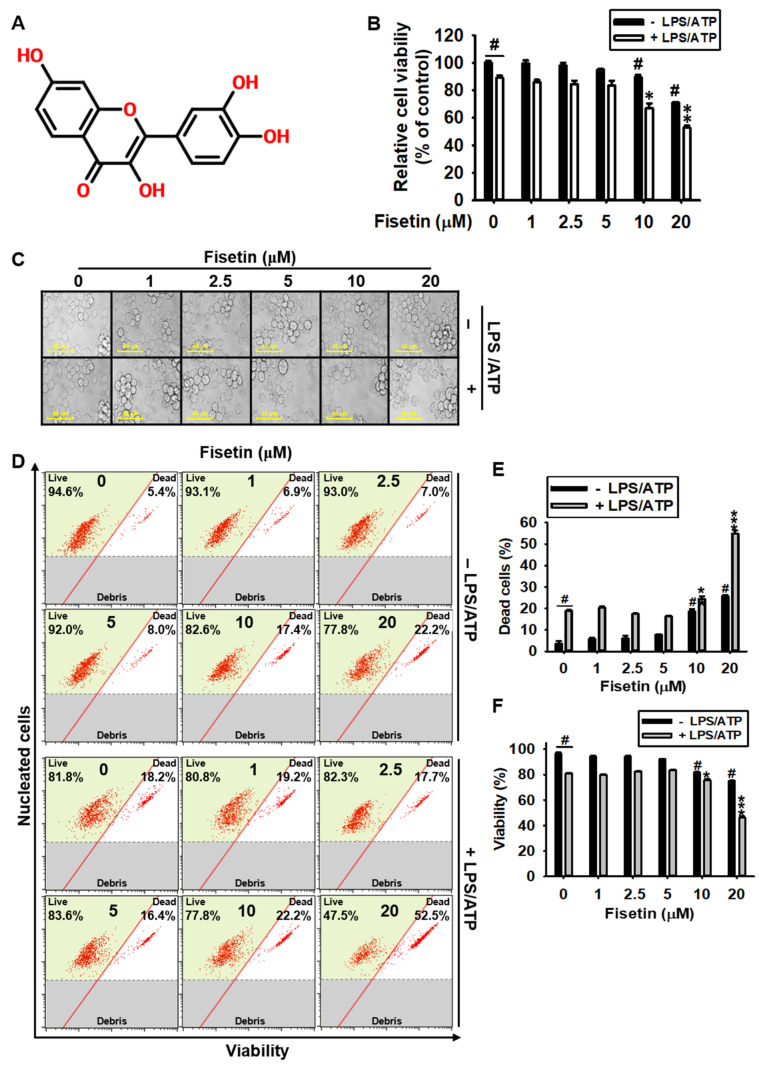
High concentrations of fisetin are cytotoxic to BV2 microglial cells. (**A**) Chemical structure of fisetin. BV2 microglial cells were treated with the indicated concentrations of fisetin (0–20 µM) for 2 h in the presence or absence of 1 µg/mL LPS for 2 h, followed by treatment with 1 mM ATP (LPS/ATP) for 24 h. (**B**) Cell viability was analyzed using an MTT assay. (**C**) Images of the cells were captured using phase-contrast microscopy (×10). (**D**) The population of dead and viable cells (%) was measured using a Muse Cell Count & Viability Kit. (**E**,**F**) Graphical representation of the population of (**E**) dead and (**F**) viable cells. The results indicate the mean ± standard error median (SEM), and is representative of the results obtained from three independent experiments. ^#^ *p* < 0.05 vs. untreated cells; *** *p <* 0.001, ** *p* < 0.01, and * *p* < 0.05 vs. LPS/ATP-treated cells.

To evaluate the cytotoxic effects of fisetin (Figure 1A), we treated BV2 microglial cells with fisetin for 24 h, in the presence or absence of LPS/ATP. We observed that cytotoxicity was determined to be 89.7% ± 1.7% and 70.9% ± 0.6% at concentrations of 10 µM and 20 µM fisetin, respectively (Figure 1B). Under LPS/ATP-treated conditions (89.2% ± 1.7%), the viability also decreased to 67.1% ± 3.4% and 52.9% ± 1.4% when treated with fisetin at concentrations of 10 µM and 20 µM, respectively. Fisetin did not show any cytotoxicity at low concentrations (≤5 μM) regardless of the presence of LPS/ATP. Treated with fisetin in the absence of LPS/ATP, it showed no marked cytotoxic phenotypes, such as cell swelling, presence of cellular debris, and floating cells (Figure 1C). Stimulation with LPS/ATP reduced the number of cells. However, the reduction in the number of cells was more pronounced when the concentration of fisetin was above 10 µM. Result of flow cytometry (Figure 1D) revealed that the population of dead cells 18.7% ± 1.1% and 25.8% ± 0.4% following treatment with fisetin at concentrations of 10 µM and 20 µM (Figure 1E), respectively, and agreed with the population of viable cells, which was 81.3% ± 1.1% and 75.5% ± 0.4% (Figure 1F), respectively, in the absence of LPS/ATP. However, fisetin augmented the cytotoxic effect of LPS/ATP, and the population of dead cells was 24.5% ± 1.2% and 54.5 ± 1.4% following treatment with fisetin at concentrations of 10 µM and 20 µM, and the population of viable cells significantly decreased to 75.5% ± 1.2% and 46.2% ± 1.4%, respectively. However, there were no alterations in the population of dead and viable cells when the concentration of fisetin was below 5 µM. Collectively, these results indicated that fisetin has no cytotoxic effects on BV2 microglial cells at concentrations below 5 µM.

### 3.2. Fisetin Binds to the TLR4/MD2 Complex and Inhibits the Downstream Signaling Pathway

**Figure 2:**
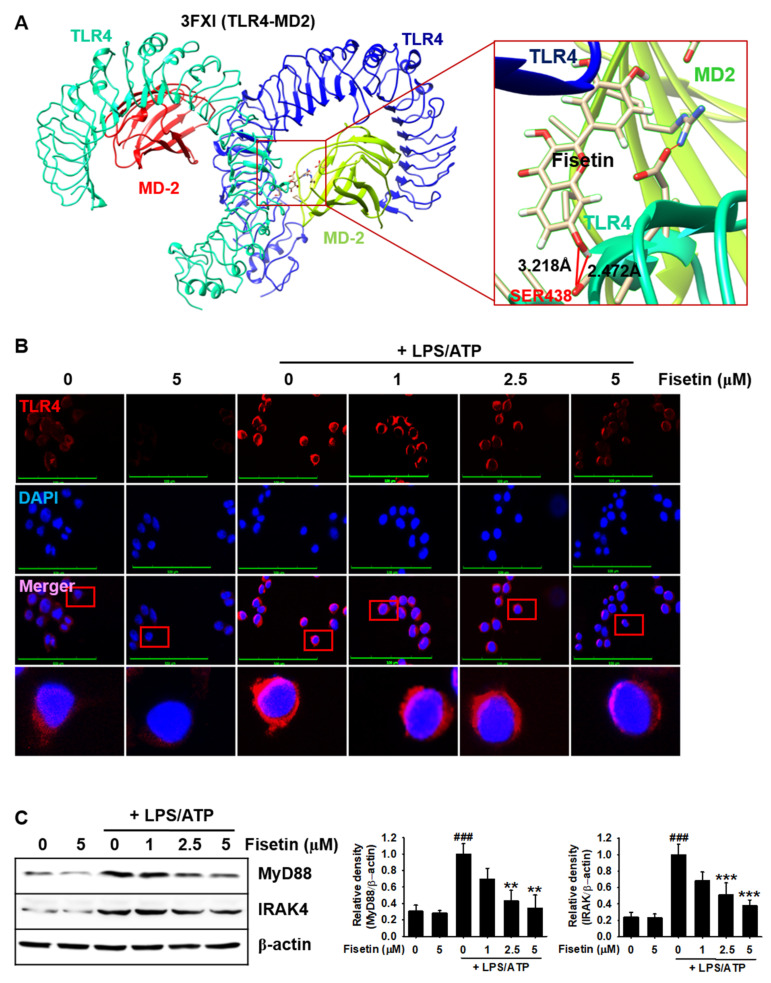
Fisetin probably binds to TLR4 and inhibits the TLR4-mediated intracellular signaling pathway. (**A**) Molecular docking of fisetin (pose 1) with the TLR4/MD2 complex (PDB ID: 3FXI). (**B**) BV2 microglial cells were seeded on 3% gelatin-coated coverslips and treated with the indicated concentrations of fisetin (0–5 µM) for 2 h prior to stimulation with 1 µg/mL LPS for 2 h and subsequent stimulation with 1 mM ATP (LPS/ATP) for an additional 2 h. Cells were fixed with 4% paraformaldehyde and immunostained for TLR4 using Alexa Fluor 647-conjugated secondary antibody. The images were captured using a CELENA S Digital Imaging System. (**C**) In a parallel experiment, the total proteins were extracted and western blotting was performed for detecting the expression of MyD88 and IRAK4 proteins. β-Actin was used as a loading control. The results indicate the mean ± standard error median (SEM), and is representative of the results obtained from three independent experiments. ^###^ *p* < 0.001 vs. untreated cells; *** *p <* 0.001 and ** *p* < 0.01 vs. LPS/ATP-treated cells.

In order to investigate whether fisetin could antagonize the TLR4/MD2-mediated signaling pathway, molecular docking prediction was used. According to the molecular docking, we predicted four binding poses of fisetin in the TLR4/MD2 complex having a strong binding affinity. The binding pose of fisetin with the strongest affinity (pose 1) showed a binding score of −6.6 to TLR4, and fisetin was shown to form hydrogen bonds with distances of 2.472 Å and 3.218 Å with SER438, which lines in the LPS-binding hydrophobic pocket of MD2 (Table 2 and Figure 2A). The three other binding poses predicted using molecular docking also bound to TLR4 and occluded the LPS-binding pocket of MD2 (Supplementary Figure S1). The binding scores and interacting residues varied among the three poses (Table 2). We next verified whether fisetin inhibits the TLR4/MD2 signaling pathway induced by LPS/ATP. Results from the immunostaining studies revealed that the fluorescence due to TLR4 was intensified by treatment with LPS/ATP, indicating that stimulation with LPS/ATP promoted the dimerization of TLR4 on the cell surface membrane. However, treatment with fisetin suppressed the intensity of the fluorescence in a concentration-dependent manner (Figure 2B). As fisetin was predicted to bind to TLR4, we investigated whether fisetin could inhibit the recruitment of intracellular adapter proteins, MyD88 and IRAK4. As expected, fisetin prevented the LPS/ATP-induced expression of MyD88 and IRAK4 in a concentration-dependent manner (Figure 2C). The results indicated that fisetin interacted with the TLR4/MD2 complex and inhibited the binding of LPS to the complex, which suppressed the LPS-induced TLR4/MD2 signaling pathway.

### 3.3. Fisetin Inhibits the NF-κB Signaling Pathway

**Figure 3:**
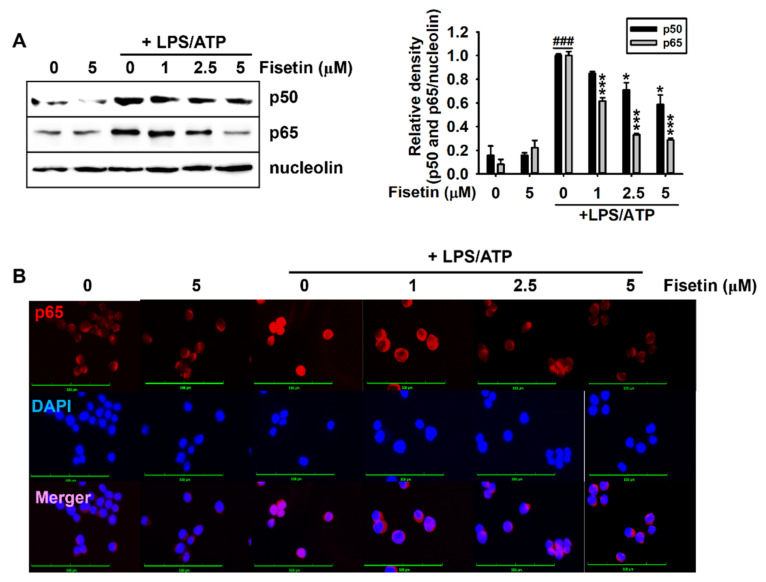
Fisetin inhibits the nuclear translocation of NF-κB. BV2 microglial cells were treated with the indicated concentrations of fisetin (0–5 µM) for 2 h and subsequently stimulated with 1 µg/mL LPS for 2 h, followed by stimulation with 1 mM ATP (LPS/ATP). (**A**) Nuclear proteins were extracted after 1 h of treatment with ATP and western blotting was performed. Nucleolin was used as a loading control. Relative densities of p50 and p65 were calculated using ImageJ software. (**B**) In a parallel experiment, the cells were fixed with 4% paraformaldehyde and immunostained for p65 with Alexa Fluor 647-conjugated secondary antibody. The images of the cells were captured using a CELENA S Digital Imaging System. The results indicate the mean ± standard error median (SEM), and is representative of the results obtained from three independent experiments. ^###^ *p* < 0.001 vs. untreated cells; *** *p <* 0.001 and * *p* < 0.05 vs. LPS/ATP-treated cells.

The nuclear translocation of NF-κB results in the transcriptional initiation of target genes, including *NLRP3*, *ASC*, *caspase-1*, and *IL-1β* [28]. Therefore, we examined whether fisetin downregulated the LPS/ATP-induced nuclear translocation of NF-κB. As depicted in Figure 3A, fisetin decreased the LPS/ATP-induced nuclear expression of the p50 and p65 subunits of NF-κB in a concentration-dependent manner (Figure 3A). Additionally, stimulation with LPS/ATP intensified the nuclear translocation of p65 (Figure 3B), which was strongly decreased by treatment with fisetin in a concentration-dependent manner. Taken together, these results indicated that fisetin could inhibit the LPS/ATP-induced nuclear translocation of NF-κB.

### 3.4. Fisetin Inhibits the Expression of the Components of the NLRP3 Inflammasome

**Figure 4:**
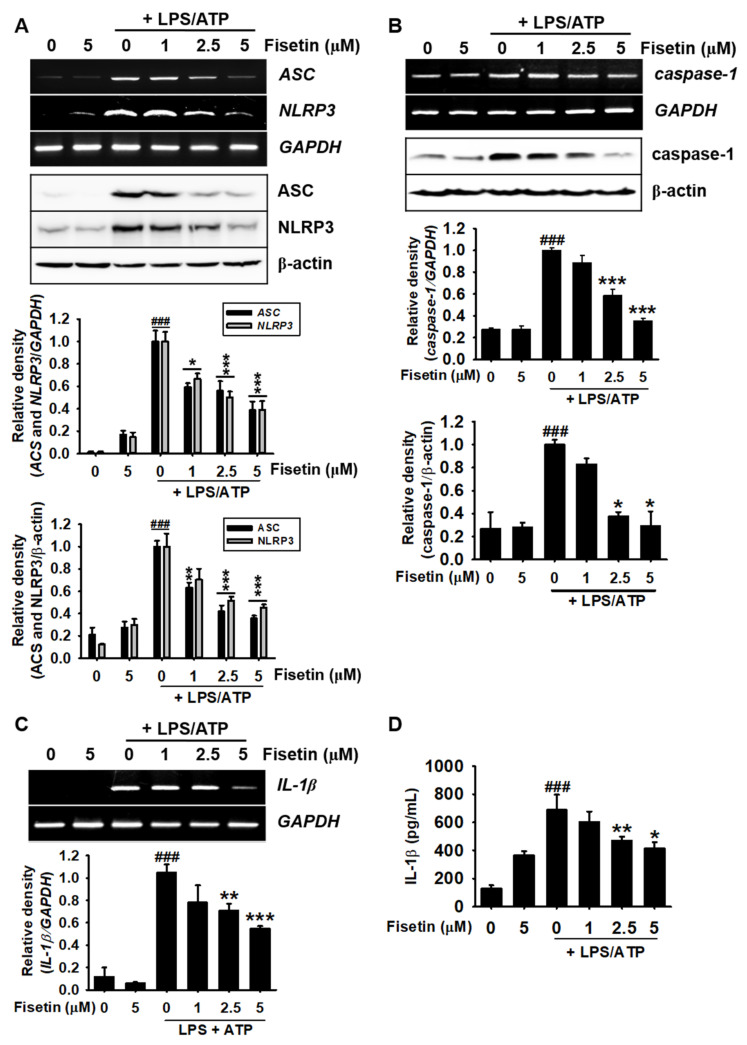
Fisetin inhibits the maturation of IL-1β by suppressing the formation of the NLRP3 inflammasome. BV2 microglial cells were treated with the indicated concentrations of fisetin (0–5 µM) for 2 h prior to stimulation with 1 µg/mL LPS for 2 h and subsequent stimulation with 1 mM ATP (LPS/ATP). (**A**–**C**) Total RNA was extracted after 9 h of treatment and total proteins were extracted after 18 h, following which RT-PCR (**top**) and western blotting (**bottom**) were performed. Relative densities were calculated using ImageJ software. (**D**) In a parallel experiment, cell culture media were collected after 48 h and the extracellular levels of IL-1β were quantified using ELISA. The results indicate the mean ± standard error median (SEM), and is representative of the results obtained from three independent experiments. ^###^ *p* < 0.001 vs. untreated cells; *** *p <* 0.001, ** *p* < 0.01, and * *p* < 0.05 vs. LPS/ATP-treated cells.

Since fisetin negatively regulate NF-κB cell signaling pathway, we assessed the effect of fisetin on the expression of the components of the NLRP3 inflammasome, including NLRP3, ASC, caspase-1, and IL-1β at the transcriptional and translational levels. The results demonstrated that the expression of both ASC and NLRP3 was upregulated at the transcriptional (Figure 4A, top) and translational (Figure 4A, bottom) levels following treatment with LPS/ATP. However, fisetin effectively inhibited the LPS/ATP-induced expression of ASC and NLRP3 in a concentration-dependent manner. Additionally, LPS/ATP-induced expression of caspase-1 was markedly inhibited by fisetin at both the transcriptional and translational levels (Figure 4B). We also measured the expression of IL-1β in the LPS/ATP-treated cells. The results demonstrated that fisetin inhibited the LPS/ATP-induced *IL-1β* expression in a concentration-dependent manner (Figure 4C). Moreover, stimulation with LPS/ATP significantly increased the secretion of IL-1β (688.2 ± 109.0 pg/mL), and treatment with fisetin inhibited the extracellular secretion of IL-1β in a concentration-dependent manner (603.6 ± 72.8 pg/mL, 469.8 ± 28.0 pg/mL, and 413.9 ± 44.3 pg/mL at 1, 3, and 5 µM, respectively; Figure 4D). These results indicated that fisetin inhibits the LPS/ATP-induced secretion of IL-1β by inhibiting the expression of the components of the NLRP3 inflammasome.

### 3.5. Fisetin Downregulates Mitochondrial Membrane Depolarization and mtROS Production

**Figure 5:**
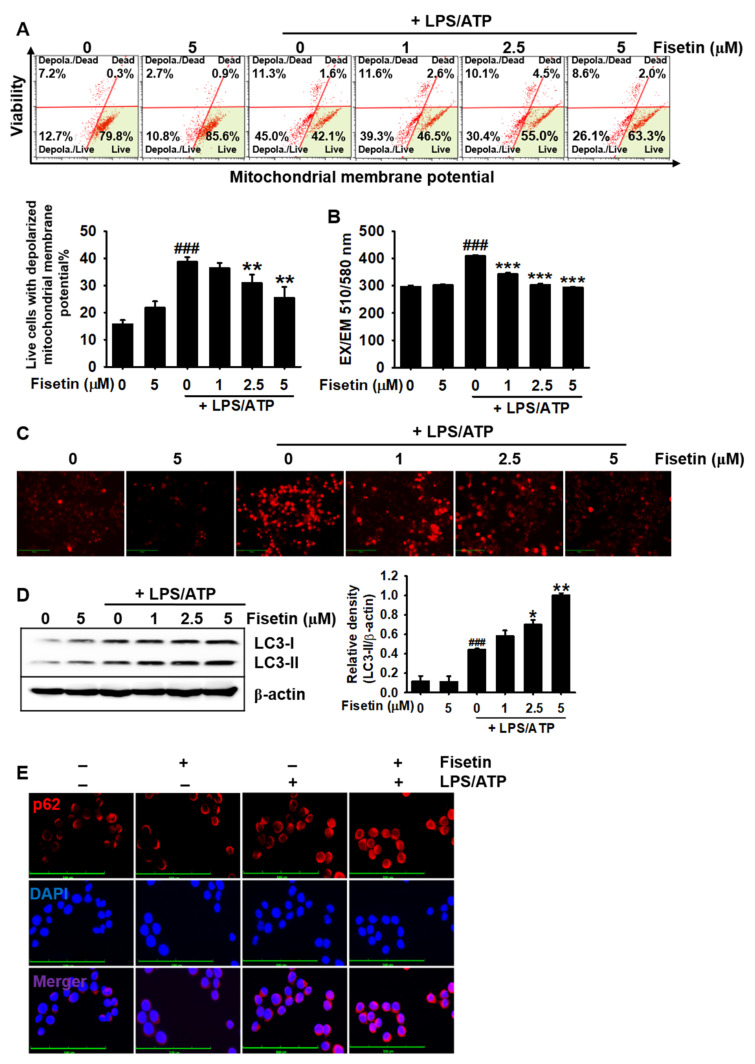
Fisetin inhibits mitochondrial membrane depolarization and promotes mitophagy. BV2 microglial cells were treated with the indicated concentrations of fisetin (0–5 μM) for 2 h and subsequently stimulated with 1 µg/mL LPS for 2 h, followed by stimulation with 1 mM ATP (LPS/ATP) for an additional 2 h. (**A**) Populations of depolarized mitochondria were measured using a Muse MitoPotential Kit. (**B**,**C**) Cells were stained with 2 µM MitoSOX Red for 10 min and (**B**) fluorescence intensities were measured using fluorometry. (**C**) Images of cells were captured using a CELENA S Digital Imaging System. (**D**) In a parallel experiment, total proteins were extracted after 9 h of treatment with LPS/ATP. Western blotting was performed for detecting the expression of LC3. β-Actin was used as a loading control. (**E**) Cells were fixed with 4% paraformaldehyde and immunostained for p62 with Alexa Fluor 647-conjugated secondary antibody. The results indicate the mean ± standard error median (SEM), and is representative of the results obtained from three independent experiments. Images were captured using a CELENA S Digital Imaging System. ^###^ *p* < 0.001 vs. untreated cells; *** *p <* 0.001, ** *p* < 0.01 and * *p* < 0.05 vs. LPS/ATP-treated cells.

Damaged mitochondria upregulate the formation of the NLRP3 inflammasome complex by inducing the excessive production of mtROS [29]. Therefore, the elimination of damaged mitochondria can be a promising strategy for preventing NLRP3 inflammasome-mediated inflammation. Therefore, we hypothesized that fisetin could inhibit the formation of the NLRP3 inflammasome by eliminating damaged mitochondria. To confirm our hypothesis, we measured the mitochondrial membrane potential (Figure 5A, top). The results indicated that stimulation with LPS/ATP increased the population of cells with damaged mitochondrial (indicated by depolarized mitochondrial membrane potential) to 41.0% ± 1.1%, whereas treatment with fisetin effectively reduced the population of cells with damaged mitochondria to 36.4% ± 2.0%, 31.0% ± 3.0%, and 25.5% ± 4.0% at concentrations of 1, 2.5, and 5 µM, respectively (Figure 5A, bottom). As the population of cells with damaged mitochondria decreased in the presence of fisetin, we analyzed the effect of fisetin on the LPS/ATP-induced production of mtROS. As depicted in Figure 5B, treatment with LPS/ATP significantly increased the intensity of MitoSOX Red from 298 ± 4.1 to 409.1 ± 3.9, and the intensity gradually decreased to 342.3 ± 5.2, 302.2 ± 6.4, and 292.5 ± 2.6 when treated with fisetin at concentrations of 1, 2.5, and 5 µM, respectively. The data obtained using fluorescence microscopy confirmed that fisetin inhibited the LPS/ATP-induced production of mtROS in a concentration-dependent manner (Figure 5C). Mitophagy plays an important role in the elimination of damaged mitochondria in cells [12]. We, therefore, investigated whether fisetin could induce the cleavage of LC3 (LC3-II), a marker of mitophagy. As depicted in Figure 5D, fisetin promoted the cleavage of LC3 in a concentration-dependent manner. The immunostaining of p62, another marker of mitophagy, confirmed that fisetin induced the expression of p62 in BV2 microglial cells (Figure 5E). These results suggested that fisetin activated mitophagy, which led to the attenuation of damaged mitochondria and the production of mtROS.

### 3.6. Transient Knockdown of p62 Reverses Fisetin-Induced Mitophagy and Formation of the NLRP3 Inflammasome

**Figure 6:**
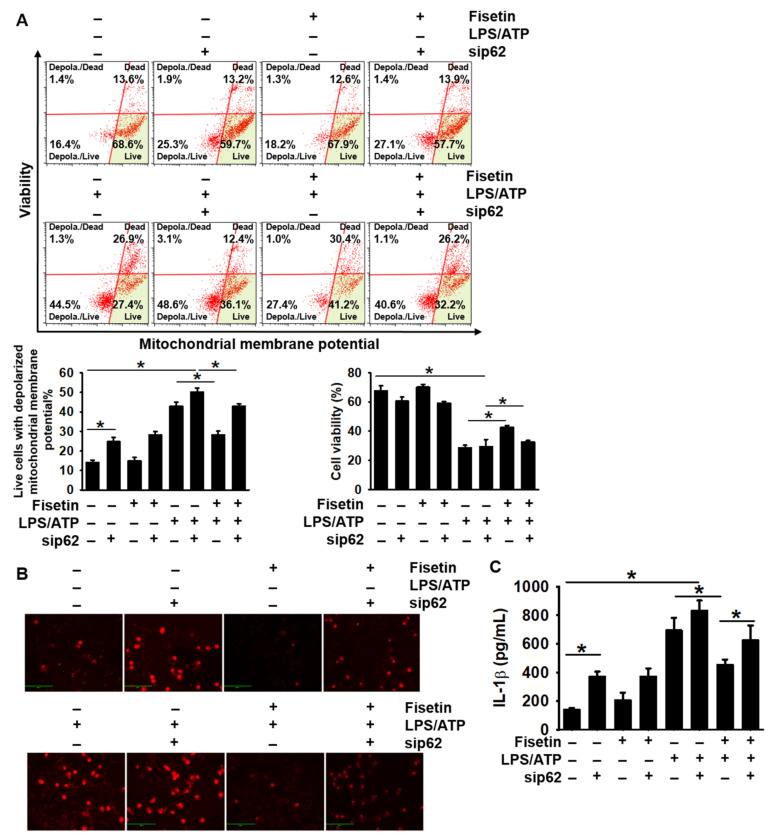
Transient knockdown of *p62* aggravates the fisetin-induced stabilization of mitochondrial membrane potential. BV2 microglial cells were transiently transfected with *p62* siRNA (sip62) for 48 h and subsequently treated with 1 µg/mL LPS for 2 h, followed by stimulation with 1 mM ATP (LPS/ATP) for 2 h. (**A**) The population of cells with depolarized mitochondrial membrane potential was measured using a Muse MitoPotential Kit. The total population of live cells with depolarized mitochondrial membrane potential (**bottom left**) and cell viability (**bottom right**) is depicted. (**B**) In a parallel experiment, cells were stained using 2 µM MitoSOX Red. Images of cells were captured using a CELENA S Digital Imaging System. (**C**) Extracellular level of IL-1β were quantified using ELISA after 48 h of stimulation of LPS/ATP. The results indicate the mean ± standard error median (SEM), and is representative of the results obtained from three independent experiments.* *p* < 0.05.

As p62 plays a pivotal role in the elimination of damaged mitochondria [13], we examined whether the expression of p62 induced by fisetin inhibits the LPS/ATP-stimulated mitochondria damage and mtROS production. As depicted in Figure 6A, the transient knockdown of *p62* increased the total population of live cells with depolarized mitochondrial membrane potential from 14.1% ± 1.1% to 24.7% ± 2.1%, compared to that of the untreated cells (Figure 6A, bottom left), and slightly decreased the population of healthy cells from 67.6% ± 3.6% to 60.6 ± 2.8% (Figure 6A, bottom right). Moreover, *p62* silencing reversed the inhibitory effect of fisetin on the LPS/ATP-induced mitochondrial membrane depolarization, and the population of cells with damaged mitochondria increased from 28.3% ± 1.9% to 42.9% ± 1.2%, whereas the population of healthy cells decreased from 42.7 ± 1.2% to 32.6 ± 1.1%. Consistent with flow cytometry data, the silencing of *p62* was associated with increased production of mtROS compared to that in the untreated cells, and the antioxidant activity of fisetin was reversed in the LPS/ATP-treated cells (Figure 6B). Lastly, we investigated the extracellular secretion of IL-1β in cells with *p62* knockdown. The transient knockdown of *p62* significantly upregulated the secretion of IL-1β (369.4 ± 13.0 pg/mL) compared to that of the untreated cells (139.1 ± 13.5 pg/mL; Figure 6C). Furthermore, the increase in the levels of IL-1β following stimulation with LPS/ATP (693.2 ± 89.0 pg/mL) was strongly intensified by transfection of sip62, to 830.6 ± 72.8 pg/mL. The inhibitory effect of fisetin on the secretion of IL-1β (452.8 ± 38.0 pg/mL) in the presence of LPS/ATP was suppressed by *p62* knockdown, to 623.9 ± 104.3 pg/mL. Altogether, these results indicated that fisetin inhibits the LPS/ATP-induced maturation of IL-1β in BV2 microglial cells in a p62-dependent manner.

### 3.7. Fisetin Inhibits Activation of the NLRP3 Inflammasome in Zebrafish Larvae

**Figure 7:**
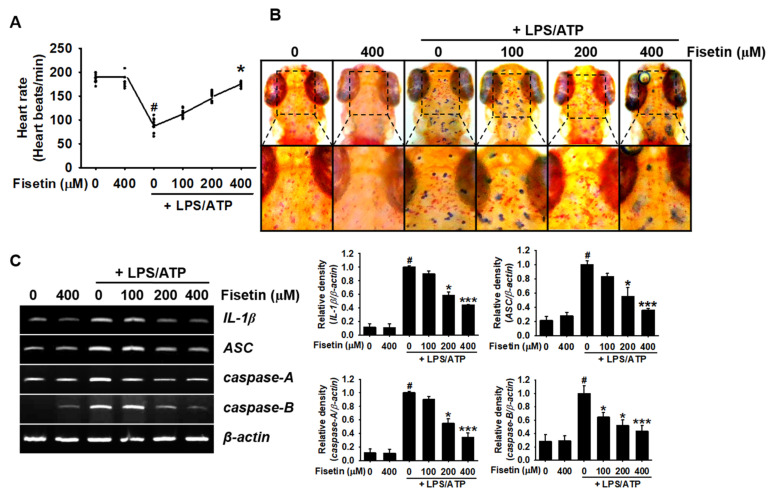
Fisetin inhibits the formation of NLRP3 inflammasome and the expression of IL-1β in zebrafish larvae. After 3 days of fertilization, zebrafish larvae (*n* = 20) were pretreated with the indicated concentrations of fisetin for 2 h prior to stimulation with 5 µg/mL LPS for 2 h, and subsequent stimulation with 2 mM ATP (LPS/ATP). (**A**) After 24 h, heart rates were measured for 2 min and expressed as beats/min. (**B**) Neutral red staining for detecting the distribution of macrophages after 24 h of treatment with LPS/ATP. (**C**) Total RNA was extracted after 18 h of treatment with LPS/ATP, and the expression of *IL-1β*, *ASC*, *caspase-A*, and *caspase-B* was determined using RT-PCR. *β-Actin* was used as a loading control. Relative densities were calculated using ImageJ software. The results indicate the mean ± standard error median (SEM), and is representative of the results obtained from three independent experiments.^#^ *p* < 0.05 vs. untreated zebrafish larvae; *** *p <* 0.001 and * *p* < 0.05 vs. LPS/ATP-treated zebrafish larvae.

To determine the inhibitory effect of fisetin on the NLRP3 inflammasome in zebrafish larvae, the larvae were treated with the indicated concentrations of fisetin (0–400 µM) for 2 h prior to treatment with LPS/ATP for 24 h. No larval deaths were observed following stimulation with LPS/ATP. However, the heart rate of the treated larvae was significantly lower (88.4 ± 4.3 beats/min) than that of the untreated zebrafish larvae (185.5 ± 2.4 beats/min, Figure 7A). The reduction in the heart rate induced by LPS/ATP significantly recovered following treatment with fisetin in a concentration-dependent manner (148.7 ± 3.0 and 173.6 ± 1.7 beats/min at concentrations of 200 µM and 400 µM, respectively). The heart rate of the larvae treated with the highest concentration of fisetin used in this study was comparable to that of untreated zebrafish larvae. Macrophage staining studies also demonstrated that treatment with LPS/ATP increased the density of macrophages in the brain compared to that of the untreated zebrafish larvae, and fisetin decreased the migration of macrophages to the brain (Figure 7B). Additionally, fisetin inhibited the expression of the genes related to the NLRP inflammasome, including *IL-1β*, *ASC*, *caspase-A*, and *caspase-B*, in a concentration-dependent manner. These results suggested that fisetin inhibits the LPS/ATP-induced formation of the NLRP3 inflammasome in zebrafish larvae, which agrees with the downregulated migration of macrophages to the brain.

## 4. Discussion

**Figure 8:**
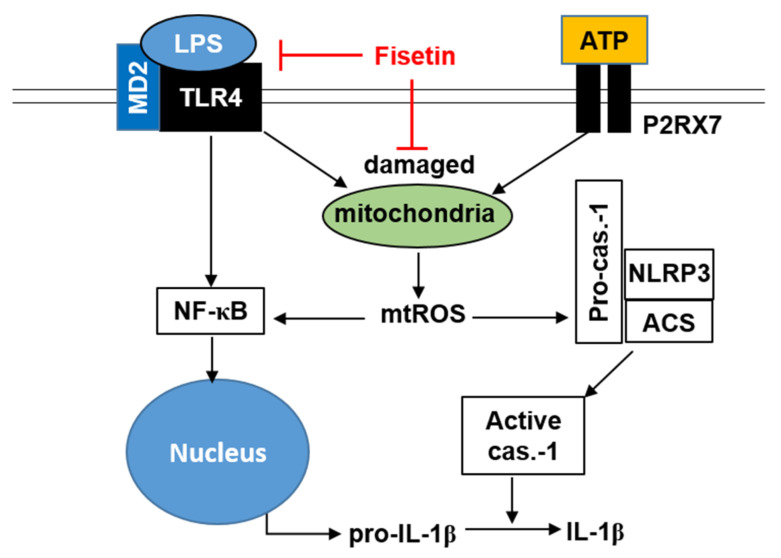
Schematic representation of the inhibitory effect of fisetin on the NLRP3 inflammasome in BV2 microglial cells. Fisetin inhibits the formation of the NLRP3 inflammasome primarily via two signaling pathways. In the first signaling pathway, fisetin competitively antagonizes the recognition of LPS by the TLR4/MD2 complex by binding to SER438 of TLR4 and occluding the hydrophobic pocket of MD2. This inhibits the canonical NF-κB signaling pathway, which consequently suppresses the transcription of *IL-1β*. In the second pathway, fisetin downregulates the production of mtROS by promoting the elimination of damaged mitochondria in a p62-dependent manner. The inhibition of mtROS production is associated with a decrease in the formation of the NLRP3 inflammasome, which subsequently inhibits the caspase-1-mediated cleavage of pro-IL-1β to active IL-1β. P2RX7, P2X purinoceptor 7.

Fisetin is a flavonol that is ubiquitous in strawberries, apples, grapes, and onions, and possesses anti-inflammatory, anticancer, and antioxidative properties [30,31]. However, the effects of fisetin on the formation and activation of the NLRP3 inflammasome have not been elucidated to date. In this study, we observed that fisetin inhibited the formation and activation of the NLRP3 inflammasome by inhibiting the TLR4/MD2 signaling pathway. We also observed that p62-meditated mitophagy plays a crucial role in the inhibition of the NLRP3 inflammasome by fisetin (Figure 8).

The NLRP3 inflammasome provides a critical molecular platform for the maturation of IL-1β, which triggers the innate immune response required for protecting the body from various pathogens and host-derived danger signals [32]. However, the aberrant activation of the NLRP3 inflammasome leads to pathological inflammatory disorders, including AD, PD, atherosclerosis, arthritis, and cancer [3,4,33]. Recently, the existence of inflammasomes in non-mammalian species including zebrafish have been identified [34]. Previous studies of Li et al. [35] suggested that the overall structural architecture of NLRP3 inflammasome in zebrafish is shared with mammalian NLRP3s, which enables the activation of classical inflammasome complex assembly to cleave the IL-1β through caspase-1 activation. Therefore, zebrafish was used as the model organism for this study. Previous studies have demonstrated that the induction of the NF-κB signaling pathway by LPS significantly increases the maturation of IL-1β by promoting the formation of the NLRP3 inflammasome [36]. Therefore, inhibition of the TLR4/MD2 complex is considered to be a promising therapeutic strategy for ameliorating NLRP3-mediated inflammatory disorders [26,37]. A study demonstrated that an intravenous injection of fisetin alleviated acute lung injury in LPS-treated mice by inhibiting the expression of TLR4 and NF-κB [17]. Although the inhibitory effect of fisetin on the expression of TLR4 and NF-κB is firmly established, there are no reports on whether fisetin directly binds to the TLR4/MD2 complex to inhibit the NF-κB signaling pathway. Interestingly, in this study, we observed that fisetin likely binds to TLR4 and occludes the hydrophobic pocket of MD2, which prevents the dimerization of the TLR4/MD2 complex by inhibiting the recognition of LPS. In a previous study, the use of MD2 mimetics was shown to inhibit LPS-induced downstream signaling by preventing the dimerization of the TLR4/MD2 complex, which in turn inhibits the MyD88/IRAK4-NF-κB axis [38]. In this study, we also observed that the inhibition of the TLR4/MD2 signaling pathway by fisetin downregulated the expression of MyD88 and IRAK4, which subsequently inhibited the nuclear translocation of NF-κB. The inhibition of NF-κB by fisetin may downregulate the expression of the components of the NLRP3 inflammasome and pro-*IL-1β*. Interestingly, Yang et al. [39] demonstrated that amyloid-β aggregates bind to TLR4 and stimulate the complement-mediated activation of the NLRP3 inflammasome, which leads to AD. We have previously demonstrated that fisetin can bind to GSK-3β, another molecular target for neuronal disorders, and induce the liberation of β-catenin [20]. This finding indicates that fisetin can target dual molecules, such as TLR4 and GSK-3β, and may be a promising therapeutic agent for neuronal disorders. Previous studies have demonstrated that the NLRP3 inflammasome also activates NF-κB by interacting with mitochondrial antiviral signaling protein and receptor-interacting-serine/threonine-protein kinase 2 [40,41]. Therefore, further studies are necessary in this regard for evaluating whether fisetin directly inhibits the formation of the NLRP3 inflammasome by acting as an upstream molecule.

Several mechanisms have been proposed to explain the events that lead to the assembly of the NLRP3 inflammasome complex. In particular, the excessive production of mtROS from damaged mitochondria is a key regulator of the assembly and activation of the NLRP3 inflammasome [8,10,14,15]. This observation is further strengthened by the fact that the NLRP3 inflammasome localizes in the vicinity of mitochondria [42], allowing the mtROS-induced assembly of the NLRP3 inflammasome. On the other hand, Yu et al. [43] demonstrated that the knockout of *NLRP3* and *caspase-1* inhibited the production of mtROS in mice in response to treatment with ATP and nigericin, indicating that the activation of the NLRP3 inflammasome activates a positive regulatory loop that perturbs mitochondrial physiology. These studies demonstrate the occurrence of crosstalk between the NLRP3 inflammasome and mtROS and suggest that targeting mtROS by the elimination of damaged mitochondria could be a promising therapeutic strategy for NLRP3 inflammasome-related immune disorders. Interestingly, a previous study demonstrated that fisetin prevents the generation of intracellular ROS in osteoclast differentiation [44] and cardiac cell death [45], indicating its antioxidative effects in various diseases. In this study, fisetin inhibited the LPS/ATP-induced activation of the NLRP3 inflammasome by inhibiting the depolarization of mitochondrial membrane potential and subsequent production of mtROS. These data indicated that the antioxidative activity of fisetin attenuated the formation and activation of the NLRP3 inflammasome.

The p62 protein is an LC3-binding cargo protein that transfers damaged mitochondria to the autophagosome, subsequently leading to their degradation [13]. A previous study demonstrated that silencing of *p62* in THP-1 macrophages elevates the LPS/ATP-mediated activation of the NLRP3 inflammasome and subsequent IL-1β maturation [46]. Another study reported that the formation of inclusion bodies is suppressed in *p62^−/−^* mice, compared to that in the wild-type mice, resulting in autophagy deficiency [47]. In this study, we observed that the transient siRNA-mediated knockdown of *p62* elevated the depolarized mitochondrial membrane potential and the production of mtROS. The transient knockdown of *p62* additionally reversed the inhibitory effect of fisetin on the formation and activation of NLRP3 inflammasome complex by inhibiting mitophagy. The elimination of damaged mitochondria can have positive cellular effects as it inhibits the assembly and activation of NLRP3 inflammation by suppressing excessive mtROS production. However, it may result in other effects, as mitochondria are closely related to cellular energy metabolism. Therefore, further studies are necessary for understanding the role of the NLRP3 inflammasome from the perspective of energy metabolism in relation to mtROS production.

## 5. Conclusions

In this study, we demonstrated that fisetin binds to the TLR4/MD2 complex and subsequently inhibits the NF-κB signaling pathway. The results further indicated that fisetin stabilized the LPS/ATP-induced depolarization of the mitochondrial membrane potential and downregulated mtROS production by activating p62-mediated mitophagy, resulting in the inhibition of the NLRP3 inflammasome. Thus, we propose that fisetin could serve as a potential therapeutic agent for inflammatory disorders induced by the NLRP3 inflammasome. Nevertheless, further preclinical trials are necessary to ascertain whether fisetin can downregulate NLRP3 inflammasome-induced immune disorders in humans.

## Figures and Tables

**Table 1:** Primers and PCR conditions used in this study.

Species	Gene *	Primer Sequence(5′-3′)	Accesion Number	Size	T_m_	Cycle No.
mouse	*NLRP3*	F: 5′-ATTACCCGCCCGAGAAAGG-3′	NM_001359638	141 bp	58 °C	27
		R: 5′-TCGCAGCAAAGATCCACACAG-3′				
	*ASC*	F: 5′-GCAACTGCGAGAAGGCTAT-3′	NM_023258	256 bp	58 °C	27
		R: 5′-CTGGTCCACAAAGTGTCCTG-3′				
	*IL-1β*	F: 5′-GCCCATCCTCTGTGACTCAT-3′	NM_008361	230 bp	65 °C	27
		R: 5′-AGGCCACAGGTATTTTGTCG-3′				
	*caspase-1*	F: 5′-CTGACTGGGACCCTCAAG-3′	NM_009807	529 bp	63 °C	27
		R: 5′-CCTCTTCAGAGTCTCTTACTG-3′				
	*GAPDH*	F: 5′-ACCACAGTCCATGCCATCAC-3′	NM_001289726	450 bp	63 °C	23
		R: 5′-CACCACCCTGTTGCTGTAGC-3′				
zebrafish	*IL-1β*	F 5′-TGGACTTCGCAGCACAAAATG-3′	NM_212844	149 bp	59 °C	27
		R 5′-GTTCACTTCACGCTCTTGGATG-3′				
	*ASC*	F 5′-GGCGGAATCTTTCAAGGAGC-3′	NM_131495	171 bp	58 °C	27
		R 5′-ACGCCGACCATTAAATCAGC-3′				
	*caspase-A*	F 5′-GAGAATTGTCCAGCTCTGCG -3′	NM_131505	198 bp	58 °C	27
		R 5′-GCCGGTAAGATTTGGTGTCC-3′				
	*caspase-B*	F 5′-CCTCGAGGATCTTGTGGAGT-3′	NM_152884	184 bp	58 °C	27
		R 5′-GCTTGATTTTGCGCAGTGTC-3′				
	*β-actin*	F 5′-CGAGCGTGGCTACAGCTTCA-3′	NM_131031	155 bp	61 °C	23
		R 5′-GACCGTCAGGCAGCTCATAG-3′				

Bp; base pair, T_m_; melting temperature. * NLRP3; nucleotide-binding domain. leucine-rich-containing family, pyrin domain-containing 3, ASC; adaptor protein apoptosis-associated speck-like protein containing a caspase recruitment domain, IL-1β; Interleukin 1β, GAPDH; glyceraldehyde 3-phosphate dehydrogenase.

**Table 2:** The docking of fisetin to TLR4-MD2 (PDB: 3FX1).

Binding Pose	Binding Score	Binding to TLR4	
		Binding AA ^1^	H-Bond ^2^ Distance (Å)
1	−6.6	SER438 (OG) ^3^	3.218
		SER438 (OG)	2.472
2	−6.3	LYS435 (NZ)	3.440
		SER438 (N)	2.862
		SER438 (OG)	3.250
3	−5.5	HIS431 (ND1)	2.250
		HIS456 (O)	2.608
4	−5.4	SER455 (OG)	2.932
		SER528 (OG)	2.699
		SER528 (OG)	1.863
		SER528 (OG)	3.087

^1^ A.A.; Amino acids. ^2^ H-bond; Hydrogen bond. ^3^ Binding position at each amino acid.

## Data Availability

The data presented in this study are available on request from the corresponding author. The data are not publicly available due to privacy restrictions.

## Supplementary Materials

The following are available online at https://www.mdpi.com/article/10.3390/antiox10081215/s1, Figure S1: Molecular docking poses between fisetin and TLR4/MD2 complex. (A) Pose 2, (B) pose 3, and (C) pose 4.
