# Supplementary material for: Fisetin Inhibits NLRP3 Inflammasome by Suppressing TLR4/MD2-Mediated Mitochondrial ROS Production
Source: Antioxidants (Basel). 2021 Jul 28;10(8):1215. doi: 10.3390/antiox10081215 (PMC8389007; doi:10.3390/antiox10081215)
Supplement: Supplementary file 1 [file antioxidants-1311790-supplementary.pdf › antioxidants-1311790-supplementary.pdf]

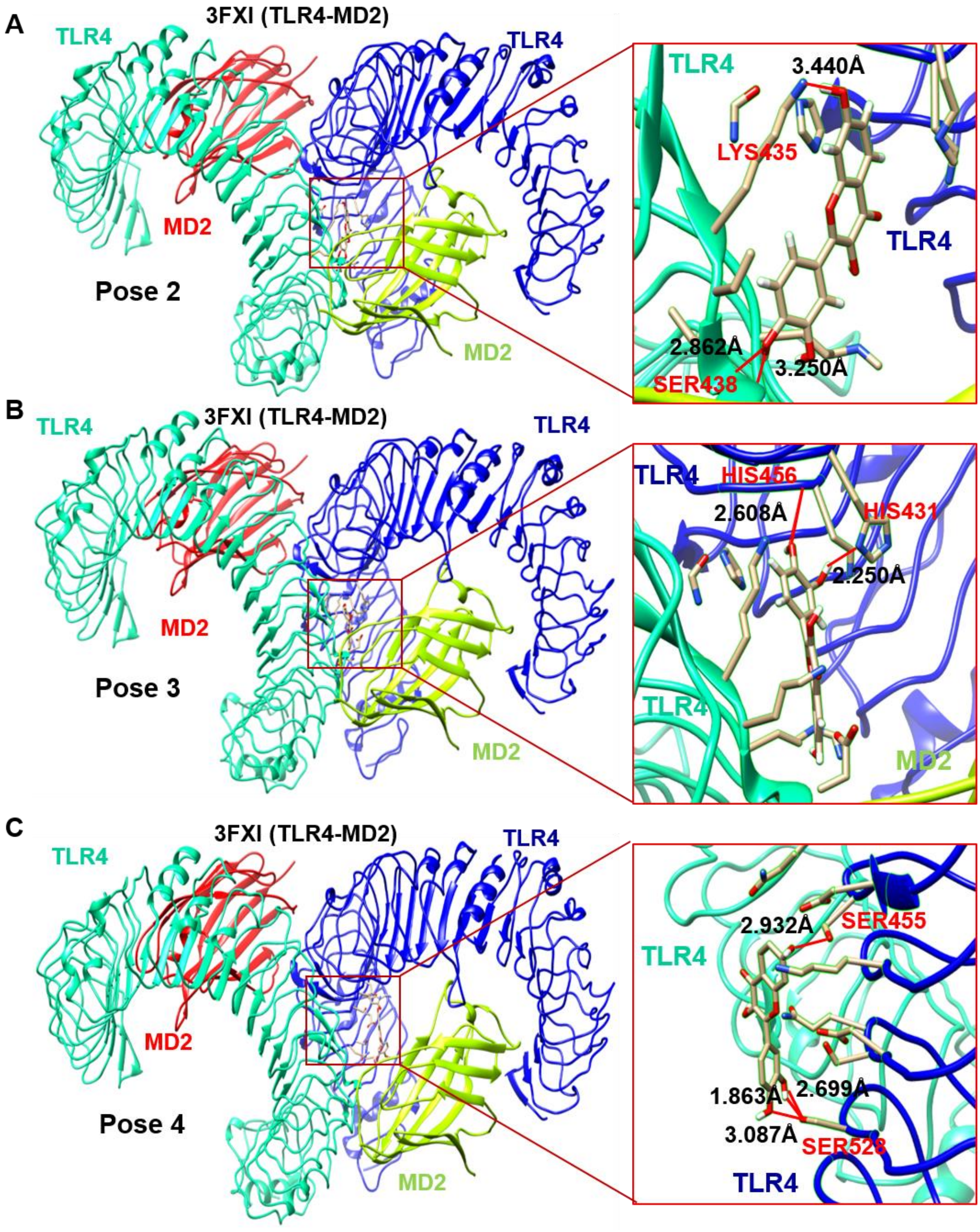

Figure S1. Molecular docking poses between fisetin and TLR4. (A) Pose 2, (B) pose 3, and (C) pose 4.
